# Inter-observer reliability assessment of the Schatzker, AO/OTA and three-column classification of tibial plateau fractures

**DOI:** 10.1186/1752-2897-7-7

**Published:** 2013-09-11

**Authors:** Yi Zhu, Cheng-Fang Hu, Guang Yang, Dong Cheng, Cong-Feng Luo

**Affiliations:** 1 Department of Orthopaedic Surgery, Shanghai Sixth People’s Hospital, Shanghai Jiaotong University, 600 YiShan Road, Shanghai 200233, China

**Keywords:** Inter-observer reliability, Tibial plateau fracture, Classification

## Abstract

**Background:**

The purpose of our study was to evaluate inter-observer reliability of the Three-Column classifications with conventional Schatzker and AO/OTA of Tibial Plateau Fractures.

**Methods:**

50 cases involving all kinds of the fracture patterns were collected from 278 consecutive patients with tibial plateau fractures who were internal fixed in department of Orthopedics and Trauma III in Shanghai Sixth People’s Hospital. The series were arranged randomly, numbered 1 to 50. Four observers were chosen to classify these cases. Before the research, a classification training session was held to each observer. They were given as much time as they required evaluating the radiographs accurately and independently. The classification choices made at the first viewing were not available during the second viewing. The observers were not provided with any feedback after the first viewing. The kappa statistic was used to analyze the inter-observer reliability of the three fracture classification made by the four observers.

**Results:**

The mean kappa values for inter-observer reliability regarding Schatzker classification was 0.567 (range: 0.513–0.589), representing “moderate agreement”. The mean kappa values for inter-observer reliability regarding AO/ASIF classification systems was 0.623 (range: 0.510–0.710) representing “substantial agreement”. The mean kappa values for inter-observer reliability regarding Three-Column classification systems was 0.766 (range: 0.706–0.890), representing “substantial agreement”.

**Conclusion:**

Three-Column classification, which is dependent on the understanding of the fractures using CT scans as well as the 3D reconstruction can identity the posterior column fracture or fragment. It showed “substantial agreement” in the assessment of inter-observer reliability, higher than the conventional Schatzker and AO/OTA classifications. We finally conclude that Three-Column classification provides a higher agreement among different surgeons and could be popularized and widely practiced in other clinical centers.

## Introduction

Tibial plateau fractures are common intra-articular injuries often leading to post-traumatic osteoarthritis. Adequate pre-operative appraisal of plain radiographs and computed tomography (CT) scans are essential for fracture classification and treatment planning. About six anatomical classification schemes are established in clinical practice. The classical ones are the OTA/AO and the Schatzker classification systems.

Recently, a new three-column classification approach was proposed by Luo [1] based on multiplanar CT images. The purpose of our study was to evaluate inter-observer reliability of the three-column classification, using the conventional Schatzker and AO/OTA system as the reference standard.

## Materials and methods

### Definitions

**Figure 1:**
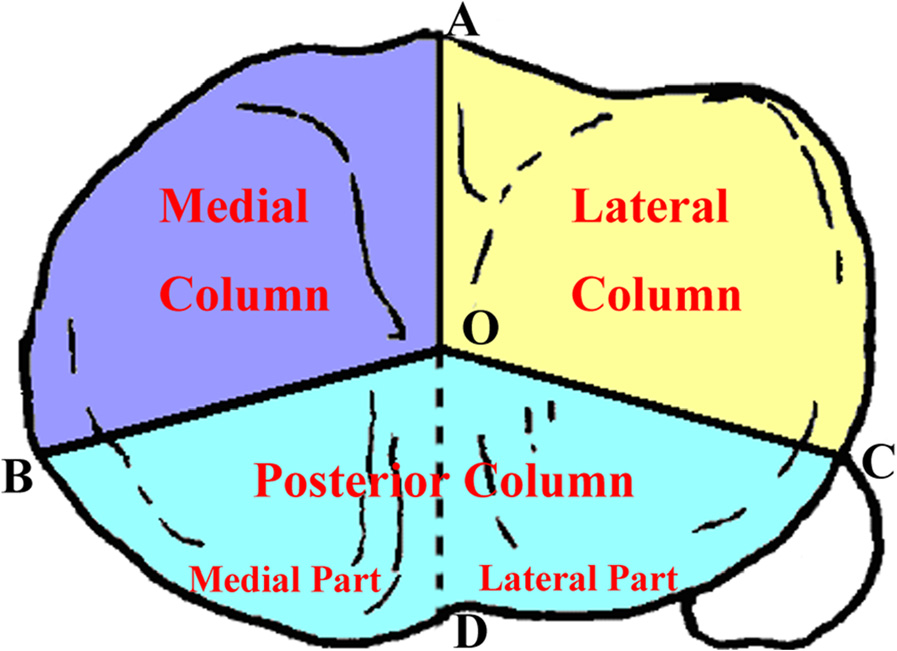
Three-Column classification.

The three-column classification of tibial plateau fractures is illustrated in Figure 1. On the transverse CT view of the tibial plateau, which firstly contains of the fibular head, the focal point (point O) is the mid-point of two tibial spines. Point A is the most anterior part of the tibial tuberosity. Point B represents the medial-posterior ridge of the tibial plateau and Point C is the most anterior point of the fibular head. The tibial plateau is divided into three parts by lines OA, OB and OC. These areas were defined as lateral column, medial column and posterior column, respectively (Figure 1). The line OD divides the posterior column into posterolateral and posteromedial parts. The rupture of the cortex wall is defined as a column fracture, which is verified by coronal and/or 3-dimensional (3-D) CT.

Articular depression with a break of the column is defined as a fracture of the relevant column. Pure articular depression (Schatzker Type III) is defined as a zero-column fracture. Most of the simple lateral split and split depression fractures (Schatzker Type I and II) belong to a one-column (lateral column) fracture. However, the concurrence of an anterolateral fracture and a separate posterior-lateral articular depression with a break of the posterior wall is defined as a two-column (lateral and posterior column) fracture. Articular depression in the posterior column with a break of the posterior wall is also defined as a one-column (posterior column) fracture (this type of fracture is not included in any type of the Schatzker classification). The other typical two-column fracture is the anteromedial fracture with a separate posteromedial fragment (medial and posterior column fracture), which traditionally belongs to Schatzker Type IV (medial condylar fracture). The three-column fracture is defined as at least one independent articular fragment in each column. The most common three-column fracture is a traditional bicondylar fracture (Schatzker Type V or Type IV) combined with a separate posterolateral articular fragment.

### Patient sample

From December 2004 to December 2006, 278 consecutive patients with tibial plateau fractures were internally fixed at the department of Orthopedics and Trauma III in Shanghai Sixth People’s Hospital. Inclusion criteria for this study were: age 18 years or older and closed fractures without neurovascular damage or evidence of compartment syndrome. Preoperative evaluation for all patients included plain radiography and computed tomography (CT) scans. 50 cases involving all fracture patterns were collected and the series was arranged randomly, numbered 1 to 50. Four observers involving one trauma surgeon specializing in the knee joint, a radiologist specializing in musculoskeletal disorders, one attending doctor and one senior resident were chosen to classify these cases; none of them was in charge of the selected patients. Before the study commenced, each observer completed a classification training session. They were given as much time as they required evaluating the radiographs accurately and independently. The observers indicated their choices on pre-designed diagrams with a schematic representation of the Schatzker and AO/OTA classification. Classifications choices had to be made at the first viewing and were not provided with any feedback. Ethical approval was not required by our institution for this type of study at the time performed.

The kappa statistic was used to analyze inter-observer reliability of the three fracture classification made by the four observers. The kappa is a chance-corrected measure of agreement comparing the observed measure of agreement with the level of agreement expected by chance alone. The guidelines proposed by Landis and Koch were used to categorize the levels of reliability based on the kappa values. Common categories are: slight agreement (0.01–0.20), fair agreement (0.21–0.40), moderate agreement (0.41–0.60), substantial agreement (0.61–0.80) and almost perfect agreement (>0.81). All statistical analyses were performed with SPSS 12.0 for Windows.

## Results

The mean kappa values for inter-observer reliability regarding Schatzker classification systems was 0.57 (range: 0.51–0.59), representing “moderate agreement”. The mean kappa values for inter-observer reliability regarding the AO/OTA classification systems was 0.62 (range: 0.51–0.71) representing “substantial agreement”. The mean kappa values for inter-observer reliability regarding the new three-column classification system was 0.77 (range: 0.71–0.89), representing “substantial agreement” (Table 1).

**Table 1:** Kappa coefficients for the inter-observer reliability of the Schatzker, AO/OTA and three-column classification

	**Schatzker**	**AO/OTA**	**Three-column**
Observer 1/2	0.576	0.710	0.731
Observer 1/3	0.582	0.635	0.706
Observer 1/4	0.570	0.603	0.890
Observer 2/3	0.513	0.586	0.721
Observer 2/4	0.589	0.510	0.730
Observer 3/4	0.572	0.692	0.818
Mean	0.567	0.623	0.766

## Discussion

A useful classification system in orthopedic trauma must 1. reliably categorize the fracture type, 2. facilitate communication in clinical practice, 3. guide preoperative planning and 4. enable comparing results and outcomes across studies. Nowadays, the most sommon classification systems used in tibial plateau fractures include Schatzker [2], Hohl and Moore [3,4], and OTA/AO system [5,6]. Charalambous et al. [7] proposed a new classification for tibial plateau fractures based on the number of condyles involved and the presence of a split/articular surface depression combination. However, all established classification schemes are not always helpful for planning the surgical strategy.

Reliability plays an essential role in the validation of a new classification. There are many comparisons among the current classifications of tibial plateau fractures. Walton et al. [8] concluded that the AO classification is superior to the Schatzker classification with regard to inter-observer reliability and should be used as a standard in scientific publications and by professional bodies. In contrast, Charalambous noted high inter-observer variability for both the Schatzker and AO/OTA classification [7].

Apparently, dividing tibial plateau fractures into unicondylar or bicondylar fractzures, and those with a pure split or articular depression with or without split may be a more reliable approach. Recently, several studies concluded that a multiplanar CT scan enhances the reliability in classifying tibial plateau fractures [9-12]. Compared to the conventional AO and Schatzker classification, a CT-based three-column classification as applied in our study is associated with substantial agreement amongst independent observers [8]. This may contribute to a more accurate assessment of fractures.

The new CT-based classification scheme allows for a distinct description of tibial plateau fractures including complex injuries. For instance, fractures extending to the posterior column are gaining more and more attention by surgeons. They are prevalent in most complex bicondylar tibial plateau fractures as a result of high-energy injury. Resulting comminution, especially with fractures involving the posterior aspect of the tibial plateau, makes the interpretation of fracture patterns difficult and prone to misconception.

Another notable injury pattern of the medial plateau is a coronal plane fracture that results in a separate posteromedial intra-articular fragment of variable size. This fragment was described in case reports in association with bicondylar tibial plateau fracture patterns and in conjunction with other proximal tibial fracture variants. Among 111 bicondylar tibial plateau fractures evaluated by plain radiographs, Higgins et al. [13] observed a 59% prevalence of a posteromedial fragment, involving about 25% of the tibial plateau joint surface. Furthermore, the posteromedial fragment was much more common and accounted for a more significant portion of the joint surface than previously described. Failure to address this fragment may allow the medial femoral condyle to rotate and dislocate posteriorly causing instability, pain, and progressive joint degeneration.

Barei et al. [14] found that 42 of 57 injuries showed a posteromedial fragment, comprising a mean of 58% of the articular surface of the medial tibial plateau (range, 19%–98%) and a mean of 23% of the entire tibial plateau articular surface (range, 8%–47%). It was emphasized that these complex interactions of fracture and implant variables should be considered by the treating surgeon when dealing with these significant injuries. Fully understanding these fractures is the basis for successful treatment. Both the Schatzker and AO/OTA systems classify these fractures according to the appearance on anteroposterior radiographs.

Thus, a classification based on the CT-scan is ultimately needed to obviate the possibilities of ignoring the posterior fragment and to design the surgical plan in a more accurate way. Brunner et al. [10] found that computed tomography scanning improves the inter- and intra-observer reliability of the OTA/AO, the Schatzker, and the Hohl classification. It was pointed out that a thorough assessment of tibial plateau fractures is a prerequisite for decision making and preoperative planning, and must include a CT-scan. This is especially valid in more complex fractures, such as those which require more detailed information regarding the localization and presence of split and depression zones or where the contra-lateral plateau has been affected. Hu [9] suggested that three-dimensional CT is a more reliable radiographic modality than 2D CT in evaluation of fracture patterns in tibial plateau fractures and supports the use of 3D-CT when analyzing complex intra-articular fractures of the tibial plateau. Eggli et al. [15] concluded that bilateral condylar tibial fractures follow a regular pattern, which is explained by the anatomic form and motion of the two tibio-femoral compartments. Existing classifications, based on 2-dimensional x-rays, do not describe these fracture types correctly and a new classification based on CT data is demanded.

A three-column fixation technique to treat multiplanar complex tibial plateau fractures, based on a three-dimensional understanding of the injuries, is recommended as a possible consequence of our research. We emphasize that this classification is most useful in high energy tibial plateau fractures.

## Conclusion

The three-column classification of tibia plateau fractures based on reformatted 3D CT scans can identify posterior column fractures and fragment likely to be missed on plain radiographs. It showed substantial and higher agreement among independent observers in than the conventional Schatzker and AO/OTA classification schemes. We suggest to implement and to validate this new system in other institutions, and to use it as a helpful tool for planning the surgical strategy.

## Competing interests

All authors certify that they have not signed any agreement with a commercial interest related to this study that would in any way limiting publication of any or all data generated for the study or to delay publication for any reason. The authors also declared that they have no competing interests.

## Authors’ contributions

CFL was in charge of the study designing. YZ carried out the reliability test and paper writing. CFH, GY and DC did the classification of the fracture cases. All the authors read and approved the final manuscript.

## References

[B1] Luo CF Sun H Zhang B Zeng BF Three-column fixation for complex tibial plateau fractures J Orthop Trauma 2010 24 683 692 10.1097/BOT.0b013e3181d436f3 20881634

[B2] Schatzker J McBroom R Bruce D The tibial plateau fracture: the Toronto experience 1968–1975 Clin Orthop Relat Res 1979 138 94 104 445923

[B3] Hohl M Tibial condylar fractures J Bone Joint Surg Am 1967 49 1455 1467 6053707

[B4] Moore TM Fracture-dislocation of the knee Clin Orthop Relat Res 1981 56 128 140 7226641

[B5] Muller MAM Schneider R Willenegger H Allgower M Patella and Tibia Manual of internal fixation 1979 New York: Springer Verlag

[B6] Muller ME NS Koch P Schatzker J The comprehensive classification of fractures in long bones 1990 Berlin: SpringerVerlag

[B7] Charalambous CP Tryfonidis M Alvi F Moran M Fang C Samarji R Hirst P Inter- and intra-observer variation of the Schatzker and AO/OTA classifications of tibial plateau fractures and a proposal of a new classification system Ann R Coll Surg Engl 2007 89 400 404 10.1308/003588407X187667 17535620 PMC1963574

[B8] Walton NP Harish S Roberts C Blundell C AO or Schatzker? How reliable is classification of tibial plateau fractures? Arch Orthop Trauma Surg 2003 123 396 398 10.1007/s00402-003-0573-1 14574596

[B9] Hu YL Ye FG Ji AY Qiao GX Liu HF Three-dimensional computed tomography imaging increases the reliability of classification systems for tibial plateau fractures Injury 2009 40 1282 1285 10.1016/j.injury.2009.02.015 19535056

[B10] Brunner A Horisberger M Ulmar B Hoffmann A Babst R Classification systems for tibial plateau fractures; does computed tomography scanning improve their reliability? Injury 2010 41 173 178 10.1016/j.injury.2009.08.016 19744652

[B11] Doornberg JN Rademakers MV van den Bekerom MP Kerkhoffs GM Ahn J Steller EP Kloen P Two-dimensional and three-dimensional computed tomography for the classification and characterisation of tibial plateau fractures Injury 2011 42 1416 1425 10.1016/j.injury.2011.03.025 21570072

[B12] te Stroet MA Holla M Biert J van Kampen A The value of a CT scan compared to plain radiographs for the classification and treatment plan in tibial plateau fractures Emerg Radiol 2011 18 279 283 10.1007/s10140-010-0932-5 21394519 PMC3139878

[B13] Higgins TF Kemper D Klatt J Incidence and morphology of the posteromedial fragment in bicondylar tibial plateau fractures J Orthop Trauma 2009 23 45 51 10.1097/BOT.0b013e31818f8dc1 19104303

[B14] Barei DP O’Mara TJ Taitsman LA Dunbar RP Nork SE Frequency and fracture morphology of the posteromedial fragment in bicondylar tibial plateau fracture patterns J Orthop Trauma 2008 22 176 182 10.1097/BOT.0b013e318169ef08 18317051

[B15] Eggli S Hartel MJ Kohl S Haupt U Exadaktylos AK Roder C Unstable bicondylar tibial plateau fractures: a clinical investigation J Orthop Trauma 2008 22 673 679 10.1097/BOT.0b013e31818b1452 18978541

